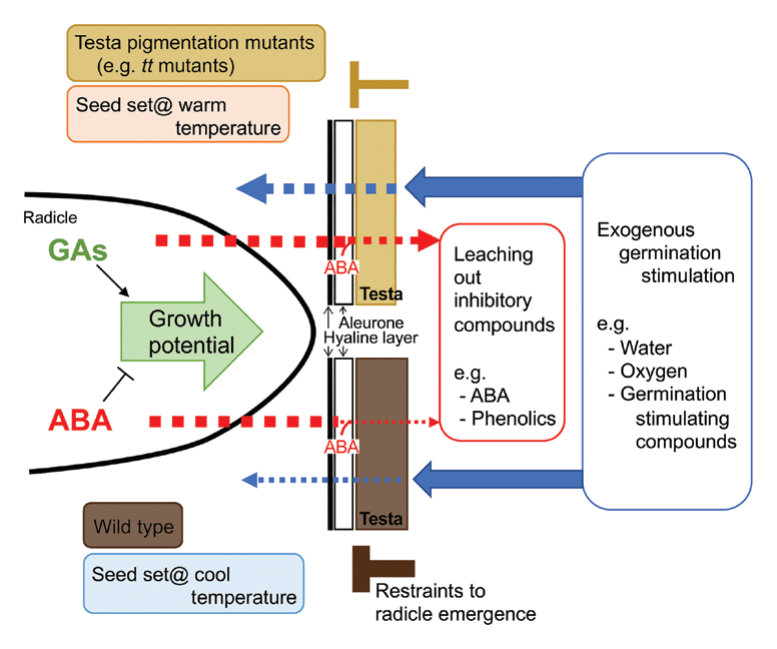# Seed dormancy and weed emergence: from simulating environmental change to understanding trait plasticity, adaptive evolution, and population fitness

**DOI:** 10.1093/jxb/erab150

**Published:** 2021-05-28

**Authors:** Kazumi Nakabayashi, Gerhard Leubner-Metzger

**Affiliations:** Department of Biological Sciences, Royal Holloway University of London, Egham, Surrey TW20 0EX, UK

**Keywords:** Agricultural weed management, climate change, dormancy trait plasticity, maternal environment, soil seed bank dynamics, weed emergence timing

## Abstract

This article comments on:

**Fernández Farnocchia RB, Benech-Arnold RL, Mantese A, Batlla D.** 2021. Optimization of timing of next-generation emergence in *Amaranthus hybridus* is determined via modulation of seed dormancy by the maternal environment. Journal of Experimental Botany **72**, 4283–4297.

**Predicting weed emergence in crop production systems is a global challenge that requires understanding mechanisms of weed ecology and trait evolution in response to climate change and altered agricultural practices. Seed dormancy is a highly adaptive trait that controls this by defining the environmental conditions in which the seed is able to germinate (Finch-Savage and Leubner-Metzger, 2006). Weed soil seed bank persistence and the timing of seedling emergence depend on dormancy (Baskin and Baskin, 2006; Walck *et al.*, 2011; Batlla *et al.*, 2020). Integrating mechanisms of seed dormancy dynamics in variable field environments and across generations with population-based models and realistic ecophysiological simulations (Fernández Farnocchia *et al.*, 2021) are essential for more sustainable weed management strategies.**

Charles Darwin wrote in his letter to Joseph Hooker (12 April 1857) ‘I have been interested in my ‘weed garden’ of 3×2 feet square: I mark each seedling as it appears, and I am astonished at number that come up.’ The timing of weed seedling emergence, often as seasonal flushes, has critical and agronomical implications as weeds produce the highest potential yield loss (30–40%) in the major crop production systems (Oerke, 2006). Weed soil seed bank dynamics depend on seed dormancy, a trait with high plasticity in weed species and with enormous adaptive value to adjust the population to a cropping system (Baskin and Baskin, 2006; Westwood *et al.*, 2018; Schwartz-Lazaro and Copes, 2019; Batlla *et al.*, 2020). The control of germination timing is achieved by seed dormancy, which can be considered as block(s) to the completion of germination of an intact viable seed under otherwise favourable conditions, namely after the seed becomes non-dormant (Finch-Savage and Leubner-Metzger, 2006). Primary dormancy is established during seed maturation prior to dispersal, whereas secondary dormancy refers to the acquisition of dormancy in a mature seed after dispersal and after the loss of primary dormancy (Graeber *et al.*, 2012; Finch-Savage and Footitt, 2017; Penfield and MacGregor, 2017). The molecular mechanisms underpinning the seasonal seed dormancy cycling to time germination in variable field environments have been investigated with *Arabidopsis thaliana* ecotypes adapted to different climates (Finch-Savage and Footitt, 2017). Seeds continually adjust their dormancy status by sensing a range of environmental signals. Temperature is related to slow seasonal change and used for temporal sensing to determine the time of year and adjust the depth of dormancy accordingly. This alters seed sensitivity to signals related to the spatial environment, including light and soil moisture. The sensing of these signals is more ultimate as they indicate if conditions are suitable for germination and therefore trigger dormancy release. Molecular mechanisms and large-scale molecular datasets of *A. thaliana* seed dormancy states (see references in Finch-Savage and Footitt, 2017) and of weed trait plasticity (Maroli *et al.*, 2018) require integration by using threshold population-based models and realistic ecophysiological simulations (Batlla and Benech-Arnold, 2014; Donohue *et al.*, 2015; Finch-Savage and Footitt, 2017). These models and simulations provide an ecophysiological framework and are especially complex if seed dormancy regulation is investigated across weed generations to capture how it maximizes weed population fitness.

Using the summer annual weed *Amaranthus hybridus* (smooth pigweed), Fernández Farnocchia *et al.* (2021) provide a sophisticated and well-integrated analysis of how the primary dormancy level at dispersal established during maturation in different maternal environments synchronizes next-generation seedling emergence timing to maximize weed population fitness. Nine *Amaranthus* (pigweed) species, including *A. hybridus*, *A. retroflexus*, and *A. palmeri*, are listed as invasive or noxious weeds, with Palmer amaranth being the most troublesome herbicide-resistant weed in south-eastern USA (Trucco *et al.*, 2009; Ward *et al.*, 2013; Assad *et al.*, 2017). Fernández Farnocchia *et al.* (2021) found that primary seed dormancy depth was lower when harvested from late season mother plants where seed maturation occurred in a short photoperiod maternal environment (Box 1). However, these observed variations in dormancy depth in the laboratory experiments did not affect seedling emergence timing in the field experiments. To interpret these results, Fernández Farnocchia *et al.* (2021) developed threshold population-based models and performed realistic simulations which generated a better ecophysiological framework for predicting seedling emergence patterns under natural conditions. Their major conclusion is that it is crucial to consider the effects of distinct maternal environments leading to variations in the depth of primary dormancy for correctly predicting weed soil seed bank dynamics, and how these contribute to the synchronization of next-generation emergence timing to maximize population fitness. Other well-investigated examples where regulation of seed dormancy by the maternal environment, in particular photoperiod and temperature during maturation (Box 2), was instrumental for maximizing population fitness in the field are *A. thaliana* (MacGregor *et al.*, 2015; Huang *et al.*, 2018; Footitt *et al.*, 2020) and the weed *Polygonum aviculare* (Batlla and Benech-Arnold, 2014; Fernández Farnocchia *et al.*, 2019; Batlla *et al.*, 2020).

Box 1. ***Amaranthus* seed structure with peripheral embryo and perisperm, and maternal effects on seed cost thickness**

The typical seed of the Amaranthaceae and of many other core Caryophyllales families evolved in the early Cretaceous and is characterized by a peripheral embryo curved around a central starchy perisperm (dead storage tissue) (Baskin and Baskin, 2019). Most *Amaranthus* species, including the weeds *A. hybridus*, *A. retroflexus*, and *A. palmeri*, and the amaranth food crops *A. caudatus* and *A. cruentus*, disperse seeds from one-seeded dehiscent fruits which open at maturity (Irving *et al.*, 1981; Trucco *et al.*, 2009; Ward *et al.*, 2013; Assad *et al.*, 2017; Ninfali *et al.*, 2020; Fernández Farnocchia *et al.*, 2021). The inner seed coat consists of a sclerified parenchyma layer with osteosclereids on either side. The outer seed coat layer of palisade sclereids can vary considerably in thickness. Fernández Farnocchia *et al.* (2021) found that the maternal environment during seed maturation on the mother plant determined seed coat thickness and depth of primary physiological dormancy. The seed coat morphological and physicochemical properties are most important for mediating the interactions between the embryo and the ambient environment. Other core Caryophyllales species with perispermic seeds disperse one-seeded indehiscent fruits (Sukhorukov *et al.*, 2015) in which the fruit coat (pericarp) properties serve this role; an example for this from the Amaranthaceae family is sugar beet (Hermann *et al.*, 2007). The maternal environment during reproduction also affects the primary dormancy depth of the dispersed fruits of the Caryophyllales (Polygonaceae) weed *P. aviculare* (Fernández Farnocchia *et al.*, 2019), but the possible effects on pericarp properties have not been investigated. The figure shows a drawing of *A. cruentus* seed structure modified from Irving *et al.* (1981), with permission from the publisher John Wiley and Sons; seed coat thickness of *A. hybridus* from Fernández Farnocchia *et al.* (2021).

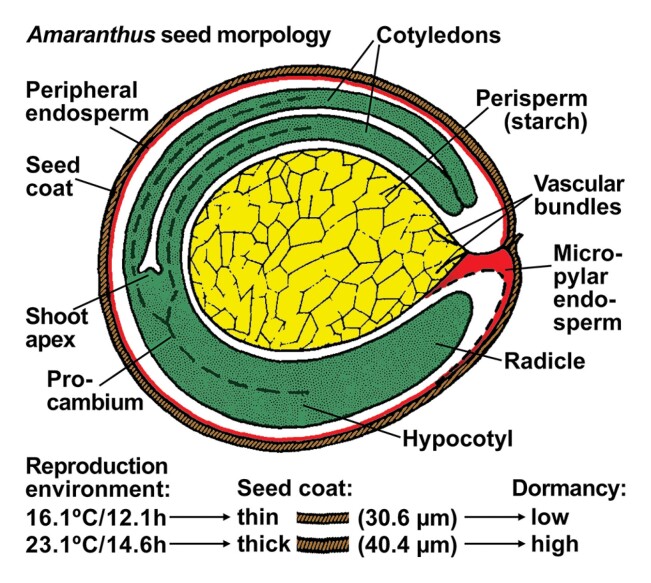

Of particular interest from a mechanistic point of view is the finding that the maternal environment, photoperiod (Fernández Farnocchia *et al.*, 2021) and temperature (MacGregor *et al.*, 2015), affected primary dormancy depths, at least in part, by altering seed coat morphological (Box 1) and physicochemical (Box 2) properties. In species with coat-imposed dormancy, the seed and fruit coat properties are a decisive component of this trait (Finch-Savage and Leubner-Metzger, 2006; Lepiniec *et al.*, 2006; Steinbrecher and Leubner-Metzger, 2017; Francoz *et al.*, 2018). In cereal grains and in *A. thaliana*, proanthocyanidins (tannins, brownish pigments) accumulate during seed coat development on the mother plant. The extent of this and thereby primary dormancy depths varies with temperature during seed production (MacGregor *et al.*, 2015), and *transparent testa* (*tt*) mutants (Debeaujon *et al.*, 2000) have reduced dormancy and altered permeability properties (Box 2). The typical seed of the Amaranthaceae (Box 1) and of many other Caryophyllales species has a peripheral embryo curved around a central starchy perisperm (dead storage tissue) evolved in the early Cretaceous (Baskin and Baskin, 2019). *Amaranthus hybridus* seed coat thickness and primary dormancy depths were affected by the reproduction environment on the mother plant (Fernández Farnocchia *et al.*, 2021). Dormancy is, however, not the only trait affected by seed coat thickness: a comparison of several weed species demonstrated that seed mortality in the soil seed bank is related to seed coat thickness (Gardarin *et al.*, 2010). In this work, the estimated annual seed mortality rates in the soil seed bank and the associated seed coat thicknesses of *A. hybridus* and *A. thaliana* were very similar, ranking in the middle tier of 18 species. Seed coats are indeed more than a protective shield formed of dead cell layers (Francoz *et al.*, 2018). They play important roles in seed germination, dormancy, longevity, and the persistence of the soil seed bank. As maternal tissues, the interaction between the mother plant’s genotype and the maternal environment during reproduction is decisive in maximizing population fitness across generations. This knowledge is required not only for developing more sustainable weed management strategies (Westwood *et al.*, 2018), but also for better understanding of the underpinning mechanisms of trait plasticity and adaptive evolution upon environmental change.

Box 2. **Physicochemical seed coat properties determine the flux of compounds required for the control of germination**

Physicochemical properties of seed and fruit coats have been shown to play important roles in the control of seed germination by providing permeability and/or mechanical restraints on germination processes (Steinbrecher and Leubner-Metzger, 2017). The outer seed coverings consist mostly of dead tissues and represent the seed’s interphase with the external environment. In addition to providing mechanical restraint, coat-associated mechanisms control or even prevent water uptake, leaching of inhibitors for embryo elongation such as abscisic acid (ABA), or gaseous exchange which may cause oxygen deficiency within the embryo. An excellent example to illustrate this are the *transparent testa* (*tt*) mutants of *Arabidopsis thaliana* which exhibit lighter testa (seed coat) colour (see figure) due to defects in flavonoid metabolism and in turn reduced proanthocyanidin biosynthesis (Lepiniec *et al.*, 2006). During *A. thaliana* seed coat development, proanthocyanidins accumulate in the endothelium, the innermost cell layer of the inner integument, while the outermost cell layer of the outer integument differentiates into mucilage-producing cells; and at seed maturity the testa consists entirely of dead tissue with oxidized proanthocyanidins as brownish pigments. In many mutants, reduced pigmentation often led to thinner testa and increased permeability for hormones or other compounds (see figure), and this was associated with reduced dormancy phenotypes of the *tt* mutants (Debeaujon *et al.*, 2000). Flavonoid biosynthesis during seed coat development was shown to be higher when seeds were matured in cool conditions (see figure), which was associated with a less permeable testa and increased primary dormancy (MacGregor *et al.*, 2015). Furthermore, the seed coat of many *tt* mutants remained permeable even when matured under low temperature. These results clearly indicate that temperature regulation for increased primary dormancy involves altering testa properties by accumulation of flavonoids. Increased permeability not only permits a greater influx of water and oxygen, but also allows leaching out of endogenous compounds which are inhibitory to germination or embryo growth (see figure). Similarly to Arabidopsis where temperature has been demonstrated to be a major factor, the maternal environmental signalling and dormancy control in *Amaranthus* seem to be an interaction between embryo and seed coat, with photoperiod during reproduction as the major factor (Fernández Farnocchia *et al.*, 2021).